# Diversity, Molecular Characterization and Expression of T Cell Receptor γ in a Teleost Fish, the Sea Bass (*Dicentrarchus labrax,* L)

**DOI:** 10.1371/journal.pone.0047957

**Published:** 2012-10-25

**Authors:** Francesco Buonocore, Rosario Castro, Elisa Randelli, Marie-Paule Lefranc, Adrien Six, Heiner Kuhl, Richard Reinhardt, Angelo Facchiano, Pierre Boudinot, Giuseppe Scapigliati

**Affiliations:** 1 Department for Innovation in Biological, Agro-Food and Forest Systems, University of Tuscia, Largo dell’Università, Viterbo, Italy; 2 Institut National de la Recherche Agronomique, Unité de Virologie et Immunologie Moléculaires, Jouy-en-Josas, Paris, France; 3 The International ImMunoGeneTics Information System®, Laboratoire d’ImmunoGénétique Moléculaire, Institut de Génétique Humaine, Centre National de la Recherche Scientifique and Université Montpellier 2, Montpellier, France; 4 Université Pierre et Marie Curie (Université Paris-06), Unité Mixte de Recherches 7211, “Integrative Immunology” Team, Paris, France; 5 Max Planck Institute for Molecular Genetics, Berlin, Germany; 6 Genome Centre at Max Planck Institute for Plant Breeding Research, Cologne, Germany; 7 Laboratory of Bioinformatics and Computational Biology – National Research Council, Istitute of Sciences of Alimentation, Avellino, Italy; 8 Centre National Recherche Scientifique, Unité Mixte de Recherches, “Immunology, Immunopathology, Immunotherapy”, Paris, France; University Paris Sud, France

## Abstract

Two lineages of T cells, expressing either the αβ T cell receptor (TR) or the γδ TR, exist in Gnathostomes. The latter type of T cells account for 1–10 % of T cells in blood and up to 30 % in the small intestine. They may recognize unconventional antigens (phosphorylated microbial metabolites, lipid antigens) without the need of major histocompatibility class I (MH1) or class II (MH2) presentation. In this work we have described cloning and structural characterization of TR -chain (TRG) from the teleost *Dicentrarchus labrax*. Further, by means of quantitative PCR analysis, we analyzed TRG expression levels both in poly I:C stimulated leukocytes *in vitro*, and following infection with betanodavirus *in vivo*. Two full length cDNAs relative to TRG, with the highest peptide and nucleotide identity with Japanese flounder, were identified. A multiple alignment analysis showed the conservation of peptides fundamental for TRG biological functions, and of the FGXG motif in the FR4 region, typical of most TR and immunoglobulin light chains. A 3D structure consisting of two domains mainly folded as beta strands with a sandwich architecture for each domain was also reported. TRG CDR3 of 8–18 AA in length and diversity in the TRG rearrangements expressed in thymus and intestine for a given V/C combination were evidenced by junction length spectratyping. TRG mRNA expression levels were high in basal conditions both in thymus and intestine, while in kidney and gut leukocytes they were up-regulated after *in vitro* stimulation by poly I:C. Finally, in juveniles the TRG expression levels were up-regulated in the head kidney and down-regulated in intestine after *in vivo* infection with betanodavirus. Overall, in this study the involvement of TRG-bearing T cells during viral stimulation was described for the first time, leading to new insights for the identification of T cell subsets in fish.

## Introduction

In vertebrates, T cells are fundamental players of the specific immune response. T cells identify the antigens through a CD3-associated, antigen (Ag) -specific and heterodimeric T cell receptor (TR) expressed on the cell surface. The majority of mature T cells display an αβ TR which recognizes peptides presented on the surface of antigen-presenting cells (APC) and reside primarily in secondary lymphoid organs. Another lineage of T cells carries a different TR composed of γ and δ TR chains. Many γδ T cells reside in epithelial layers of mucosal tissues, such as skin, intestinal epithelium, lung and tongue, where they function as first line of defense as immunity effector cells [1]–[3]. The TR γ (TRG) and TR δ (TRD) chain proteins are encoded by genes somatically rearranged through the V-(D)-J recombination process during intrathymic T cell maturation [4]. In contrast to αβ T cells, the available repertoire of γδ T cells can be rather restricted since canonical γδ TRs are expressed in specific anatomical locations [5]. The γδ T cells display cytotoxic activity [6], can lyse infected macrophages and, in this way, limit the spread of infectious microorganisms [7]. A peculiar feature of γδ T cells is that they do not recognize peptides processed from complex protein antigens by APC cells, but rather individuate unconventional antigens such as phosphorylated microbial metabolites and lipid antigens. Moreover, the presentation of these ligands by major histocompatibility class I (MH1) or class II (MH2) proteins is not needed, and this is in agreement with the absence of CD4 or CD8 expression in the majority of γδ T cells [3], [8]. For these reasons, and based on the interpretation that γδ T cells use their T cell receptor as a pattern recognition receptor, these cells have been considered, functionally, as a bridge between the innate and adaptive immune systems [9]. However, TRG and TRD genes have all the features of the rearranging genes of the adaptive immune response that characterizes vertebrates with jaws, from fish to humans [4]. An experimental 3D structure of a human γδ T cell receptor from a T cell clone that is phosphoantigen-reactive was obtained [10] and showed a peculiar orientation of the variable and constant regions in the complex when compared to αβ TR. The obtained 3D structures permitted putative structural interactions between γδ TR with CD3 isoforms and other ligands, and have been of great help for the understanding of important mechanisms necessary for receptor assembly, ligand recognition and signaling [11]–[12]. Typical TRG and TRD sequences were found in Chondrichthyans, suggesting that these genes were present in the common ancestors of all jawed vertebrates [13]. In teleost fish, TRG sequences have been identified in species like the green spotted pufferfish (*Tetraodon nigroviridis*) [14], Japanese flounder (*Paralichthys olivaceus*) [15], rainbow trout (*Onchorhynchus mykiss*) [16], and common carp (*Cyprinus carpio* L.) [17]. Moreover, several Atlantic salmon (*Salmo s*alar) TRG sequences have been identified in BAC clones [18] and, recently, a TRG locus has been sequenced in the sandbar shark (*Carcharhinus plumbeus*) showing an organization similar to that found in mammals, with a set of V genes preceding J and C genes [19]. While the biology and implication in immune defense of fish αβ T cells has been partly unrevealed, the role of γδ T cells is still unknown.

As a fish of significant importance for farming, the European sea bass (*Dicentrarchus labrax* L.) is one of the best studied teleost fish species, and much information on its immune system is available. To better understand the response to pathogens and to develop vaccination, sea bass systemic and mucosal αβ T cells have been intensely investigated in recent years. In this work, we extend these efforts to the γδ T cell immunity. We identified the sea bass TRG chain, and provided a first description of its diversity, and its 3D structure considering the putative interactions with the TRD chain. We also preliminarily investigated the modulation of TRG after *in vitro* stimulation with poly I:C and *in vivo* infection with betanodavirus.

## Results

### Sea Bass Possess Typical TRG Genes

**Figure 1:**
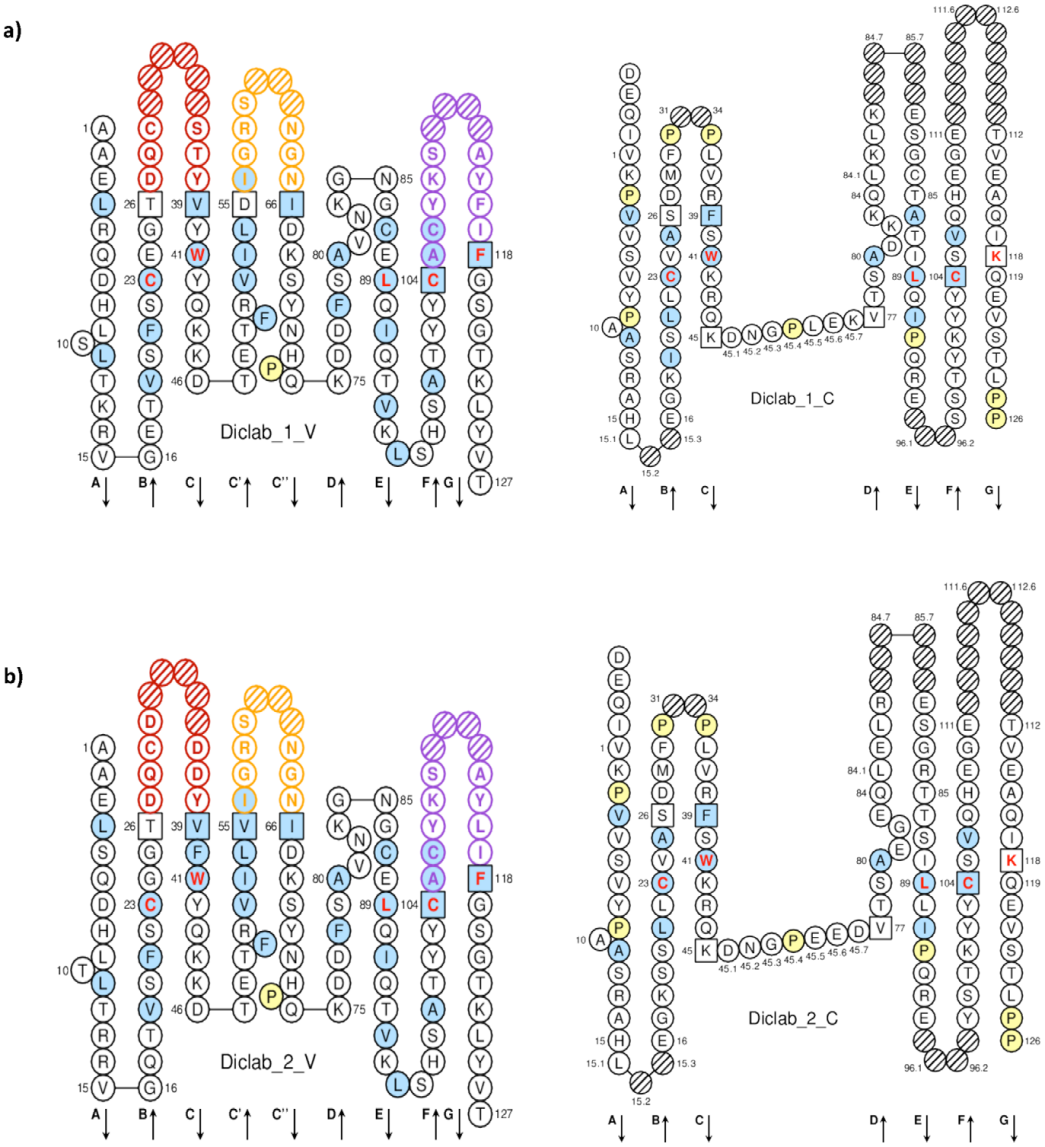
IMGT Colliers de Perles of the two identified sea bass TRG sequences. Panel a) Vdomain (V-GAMMA) and C- domain (C-GAMMA) of Diclab1, Panel b) V- domain (V-GAMMA) and C- domain (C-GAMMA) of Diclab2. Amino acid positions are according to the IMGT unique numbering for V domain and C domain. Anchor positions are shown as squares. In FR-IMGT, the hydrophobic amino acids (hydropathy index with positive value) and tryptophan (W) found at a given position in more than 50% of sequences are displayed with a blue background color.

**Figure 2:**
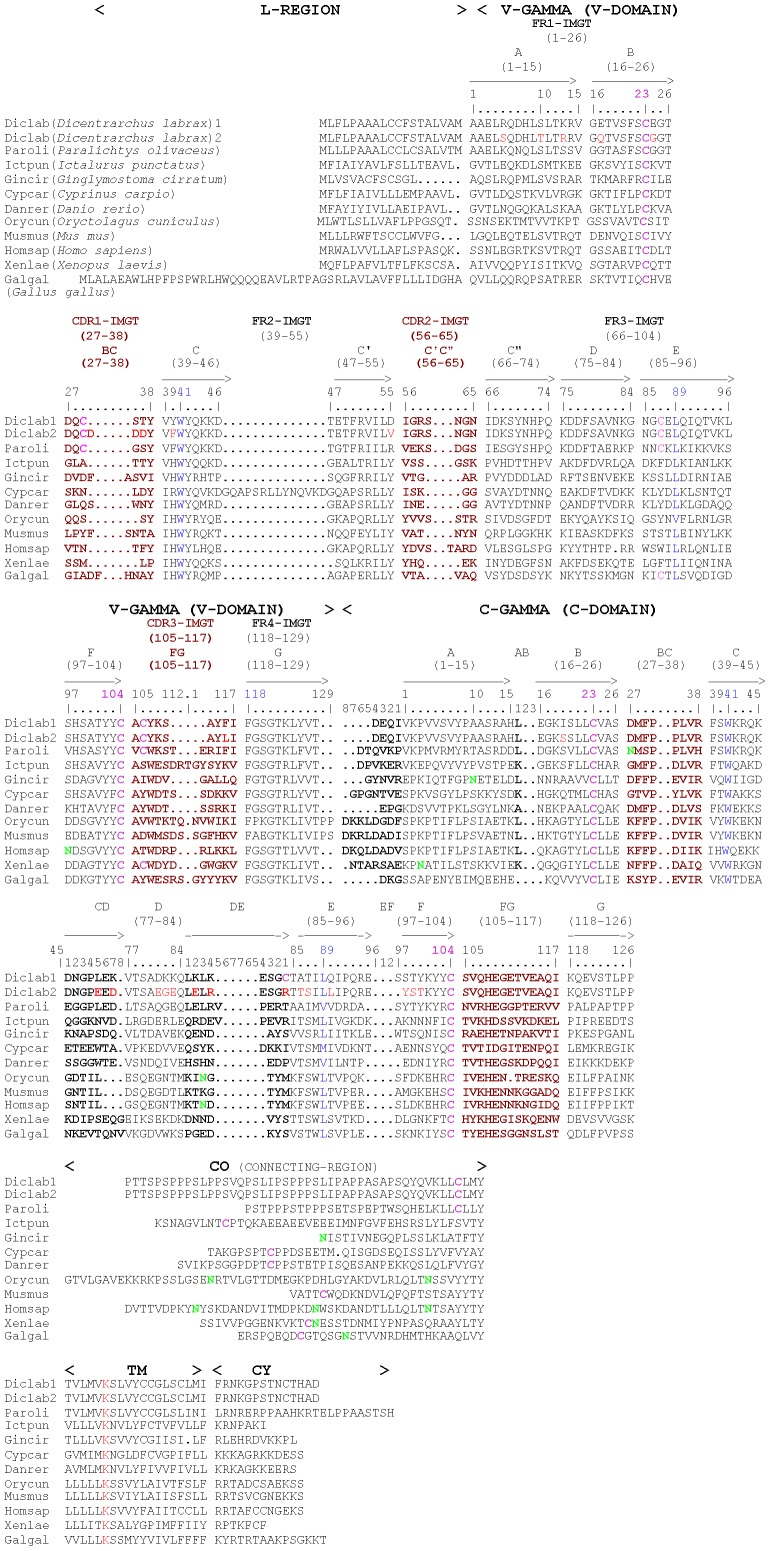
Alignment of the predicted sea bass TRγ chain amino acid sequences with other known TRγ molecules. The position of the framework (FR) and CDR regions for the V-DOMAINs following the IMGT numbering are indicated above the sequences. Conserved amino acid residues are evidenced. Accession numbers: *Dicentrarchus labrax* clone 1 FR745889; *Dicentrarchus labrax* clone 2 FR751038; *Paralichthys olivaceus* AB076073; *Ictalurus punctatus* DQ435303; *Ginglymostoma cirratum* FJ513785; *Cyprinus carpio* ABD04398; *Danio rerio* AY973921; *Oryctolagus cuniculus* RABTCRG; *Mus musculus* MUSTCRGZZX; *Homo sapiens* S01895; *Xenopus laevis* AF440821; *Gallus gallus* GGU22666.

TRG V and C sequences from human, mouse, Japanese flounder and zebrafish were used to mine sea bass ESTs and genomic resources using tblastn (Kuhl H. and Reinhardt R.**,** personal communications). Several partial sequences were identified (data not shown) and allowed the design of primers for direct or RACE amplification of sea bass TRG from the thymus. These PCR products were cloned and sequenced, and allowed the reconstitution of two full-length cDNAs corresponding to sea bass TRG (EMBL accession numbers FR745889 and FR751038). The amino acid sequences represented as “IMTG Collier de Perles” [20]–[22] (Figure 1) obtained for the two identified sea bass TRG sequences show that amino acids which are fundamental for the TRG 3D structural configuration are conserved in the two sequences. A slight difference can be noted in the two clones within the first loop of the V-DOMAIN, whereas the C-DOMAIN is well conserved. A putative 20 amino acid signal peptide was predicted in these two full length sequences (names Diclab1 and Diclab2) as well as nine O-glycosilation sites but no potential N-glycosylation. Comparison of the sea bass TRG Diclab1 nucleotide and amino acid sequence to its closest counterparts in other species is shown in Table 1. The highest nucleotide and amino acid identity was with Japanese flounder, followed by channel catfish, whilst the lowest identity was with chicken. A multiple alignment of full length sea bass TRG amino acid sequences with other known TRG sequences was assembled (Figure 2) to investigate the conservation of characteristic amino acid residues involved in structural domains. Some amino acids are conserved in all considered sequences: C^42^ (1st-CYS 23) , W^54^ (CONSERVED-TRP 41), Y^55^, Y^112^, C^114^ (2nd-CYS 104), F^124^ (J-PHE 118), G^127^, T^128^, L^130^, V^132^ in the V-DOMAIN, C^161^ (1st-CYS 23), P^168^, W^175^ (CONSERVED-TRP-41), C^221^ (2nd-CYS 104) in the C-DOMAIN, and Y^291^, K^297^ in the transmembrane region (IMGTunique numbering for V domain [23] and C domain [24] shown between parentheses). The two cysteines that in human TRG are involved in an intra-chain disulfide bond fundamental for the assembly of the V-DOMAIN [10] are conserved in Diclab1 and Diclab2, as well as the di-glycine bulge FGXG in the FR4 region, typical of most TR and IG light chains [23]. The C domain showed the conservation of the two cysteine residues involved in human TRG in the formation of another intrachain disulfide bond [10], [24], and the characteristic motif CX6PX6WX45C [17]. This motif corresponds to a BC loop of 8 amino acids long, with the conserved cysteine C23, proline P30, tryptophan W41 and cysteine C104. The connecting region (CO) showed no conserved amino acids, while in the transmembrane region (TM) a lysine K, that is important for the assembly of the complex between TR β chain and CD3 [25], is conserved in sea bass TRG sequences.

**Table 1:** Percentage of nucleotide identity and amino acid identity and similarity of sea bass TR γ Diclab1 with other known sequences.

	% Nucleotide identity	% Amino acid identity	% Amino acid similarity
*Paralichthys olivaceus*	69,8	55,6	68,8
*Ictalurus punctatus*	51,9	36,8	48,9
*Danio rerio*	50,0	35,7	48,6
*Cyprinus carpio*	48,3	34,7	48,3
*Ginglymostoma cirratum*	48,7	27,9	45,5
*Homo sapiens*	46,0	23,2	41,9
*Mus musculus*	45,9	23,0	40,1
*Oryctolagus cuniculus*	46,3	21,6	40,8
*Gallus gallus*	45,7	21,3	38,9
*Xenopus laevis*	48,8	25,3	41,6

The highest values have been evidenced in bold (for sequence accession numbers see Figure 2 legend).

**Table 2:** Sequence of primers used to study different V and C combinations.

*Name*	*Sequence (5′-3′)*	*Note*
VG1.1&2	GWTATTGGTAGGAGTAATG	W = A/T
VG1.3&4	CTTTATTTTAACAAGAACWCYTG	W = A/T, Y = C/T
VG1.5	TGATAAGAGTAATGGTCGAGTTG	
VG1.6 ext	GTGTAGTTCTTCTGTAATC	
VG1.6 int	CCAGTACATCGAGTAAGATG	
CGfluo	TATGTAACAGATGAGCAGATAGTG*	
CG1&4&5	TGTACAGCCACCATCTTGCW	W = A/T
CG2&3	CGCACCACCTCCATCTTGCT	
CG3	CAGCAGCCTCCTGTCCTCCAGAG	
CG5	CGTCTCCAACATCACTTCCA	

### V/C Combinatory Diversity of Sea Bass TRG Chain

**Figure 3:**
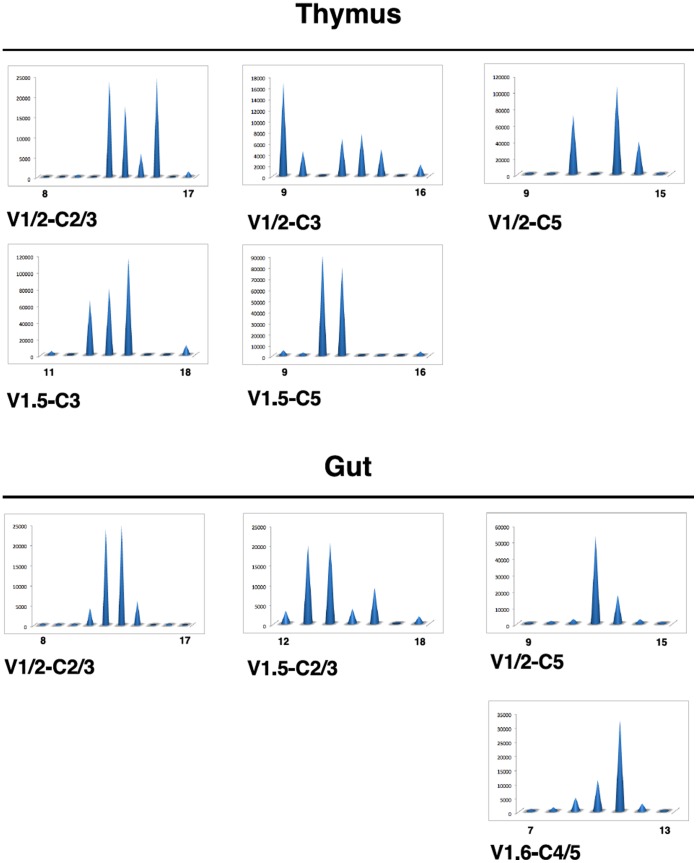
Spectratyping analysis of the γ TR diversity in gut and thymus of healthy fish. CDR3 length distribution profiles of γ transcripts for selected Vγ-Cγ combinations in naive thymus and gut. Typical results from the same fish are shown. Y-axis: fluorescence arbitrary units, x-axis: CDR3 size, with values indicated for the smallest and longest CDR3 amplified in each profile.

*In silico* mining and RACE-PCR led to the identification of several additional partial TRG sequences (Figure S1). Their comparison defines six V domains belonging to the same subgroup (Vγ1.1–1.6) and five C domains representing most likely different genes (Cγ1–5) (temporary nomenclature). To get insight into the expression and diversity of TRG transcripts combining these V and C genes, we used primers that were specific for V and C subgroups (Table 2) to test all combinations on thymus and gut cDNA from adult healthy bass. Relevant amplification was obtained for only a minority of combinations even in the thymus, suggesting that rearrangements may be restricted to recombination units as classically described for TRG in other species. These PCR products were subjected to a runoff with an internal fluorescent primer common to all Cγ sequences and the product size was resolved in a sequencing system, leading to CDR3 length spectratypes for the relevant combinations. Typical results obtained from 3 different fishes are shown in Figure 3. The profiles show a moderate but significant junction size diversity for all the combinations amplified, with two to six main peaks, in both thymus and gut. Interestingly, CDR3 distributions did not appear to be gaussian-like in the thymus for any of the amplified combinations, as typically observed for fish or mammalian TRB. Taken together, these results show that several, but not all, Vγ/Cγ transcripts are expressed in thymus and gut of adult sea bass, with various junction size distributions suggesting recombination or selection constraints.

### 3D Structure of Sea Bass TRG Chain

**Figure 4:**
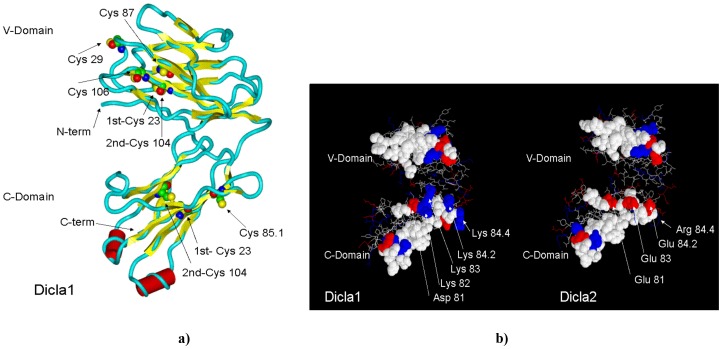
3D model of sea bass TRγ chain. Panel a). 3D model of sea bass Diclab1. The backbone ribbon and secondary structure topology are shown: yellow arrows represent β-strands and red cylinders represent α-helices. The Cys residue are evidenced along the sequence. Panel b). Amino acid residues putatively involved in the interactions with TRδ chain in both Diclab1 and Diclab2 sea bass TRγ sequences are represented as space-fill balls. The basic and acidic amino acid residues have been indicated in blue and red, respectively. Arrows and labels indicate the charged amino acids of the evidenced areas in the two sea bass TRγ sequences.

The model of sea bass Diclab1 TRG chain shows the typical fold of immunoglobulin family (Figure 4, panel a). It consists of two domains, and folded mainly as beta strands which form two beta sheets and showing a sandwich architecture for each domain. Few shorts helices are also observed. Each domain is characterized by the presence of a disulphide bond (see Figure 4, panel a), in particular 1st-CYS 23 - 2nd-CYS 104 in both domains. Four other cysteines are present in Diclab1, while in Diclab2 the last cysteine is substituted by an arginine. The conformation of the backbone suggests that they are not suitable to form other intra-chain bridges, but well exposed on the surface of the protein, so their involvement in inter-chain disulphide bridges with the TRD chain cannot be excluded.

Comparing the different TRG sequences found in amplified clones and in genomic contigs, it appeared that the sequence of a TRG V domain showed a large deletion corresponding to the region C′C′′loop (CDR2-IMTG) -C′′ strand -D strand (Figure S1). Since this deletion was found both in three different transcripts from the gut (data not shown) and from a genomic contig (contig 142010153540001), it could not be explained by a PCR artifact of by a splicing pattern using internal crypting sites as in trout Vβ6 or human NKP30 [26]–[27]. Additionally, CDR3 spectratyping confirmed that this short TRG V domain is expressed in the gut in diverse TRG V1.6/C4 or 5 rearrangements, at least at the transcript level, since a profile with 5 peaks was observed at the expected size. No profile was seen at the size predicted for a rearrangement of a TRG V1.6 sequence without deletion, which would represent a splicing variant as described for trout TR Vβ6. No amplification was observed in the thymus for any TRG V1.6/C combination. Another TRG V sequence found in a genomic contig contained a CXCW motif (IMGT position104–107) that has been found in the mouse functional TRGV5 and TRGV6 genes and in the human TRGV11 (an ORF by abscence of splicing of the leader exon) (Figure S1, sequence Part 7) (IMGT Repertoire, http://www.imgt.org, IMGT/GENE-DB [28].

### *In vitro* and *in vivo* TRG Expression Analysis

A basal expression of TRG in all examined tissues of un-stimulated sea bass was already described in [29], with the highest levels detected in thymus and intestine.

**Figure 5:**
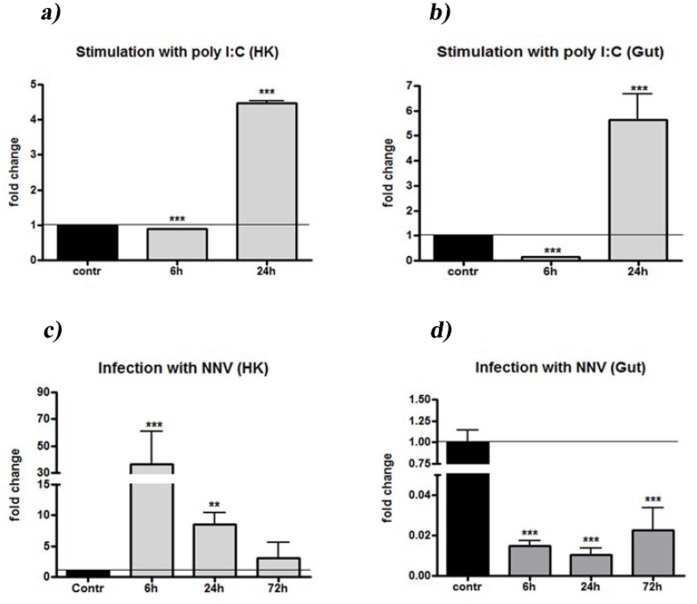
TRG expression analysis. Panel a) and b). Stimulation with poly I:C. TRγ mRNA levels expressed as a ratio relative to rRNA 18 levels in the same samples after real-time PCR analysis of head kidney and gut leukocytes stimulated with PBS (control) and with 20 µg/ml of poly I:C for 6 and 24 h and normalised against the non-stimulated 0 h control. Data are expressed as the mean ± SD; one asterisk indicates when p<0.05 with respect to the time 0 control; two asterisks indicate when p<0.01 with respect to the time 0 control and three asterisks indicates when p<0.001 with respect to the time 0 control. Panel c) and d). Infection with betanodavirus. TRγ mRNA levels as a ratio relative to rRNA 18 levels in the same samples after real-time PCR analysis of head kidney and gut leukocytes in control fish and in infected fish at different sampling times. Data are expressed as indicated above for panel a) and b).

To investigate whether TRG expression levels could be modulated by the interferon system , leukocytes from head kidney and gut were stimulated *in vitro* with the interferon modulator poly I:C for 6 or 24 hours (Figure 5, Panel a and b). In leukocytes from both tissues, a slight expression decrease was detected after 6 h, whereas a significant increase was evidenced after 24 h of stimulation (p<0,0001).

TRG expression levels were also determined in head kidney and gut from sea bass juveniles infected with betanodavirus [30], compared to non-infected controls. In the head kidney, TRG expression was highly up-regulated (30-fold) 6 hours after the infection, then decreased at 24 and 72 hours post-infection. In the intestine, down-regulation of TRG expression was obtained following viral infection at all tested time points, suggesting a possible migration of γδ T cells out of this tissue.

## Discussion

In mammals, the lymphocytes expressing αβ TR and γδ TR receptors display marked differences both in their origin and function. While αβ T cells recognize peptides presented by MH proteins, γδ T cells have a broader range of ligand type and can be cytotoxic independently from antigen presentation. The biology of fish γδ T cells remains largely unknown, although the presence of typical TRG and TRD genes in fish genomes and a few expression data indicated that they probably have a role in immune defense, especially in mucosal territories. To get insight into the γδ T cell function through vertebrates, we studied the structure, diversity and expression of the TRG receptor in a teleost fish, the sea bass.

Two full-length sea bass TRG sequences were characterized from this species, which had all the hallmarks of typical TRG sequences and were most similar to TRG genes previously identified in other fish species. The IHWY motif (IMGT positions 39–42) in the FR2 of the V domain, typical of many mammalian TRG chains [13], is changed to VYWY (found in human TRGV9) and VFWY in sea bass, as observed in Japanese flounder, whereas VHWY and IRWY are found in channel catfish and carp, respectively.

CDR3 hypervariable loop confers epitope-specific sensitivity to TR α- and β-chains, whereas CDR1 and CDR2 are mainly in contact with the MH α helices and with the ends of the peptide located in the peptide-binding groove [24], [31]–[33]. For TRG, the CDR3 loop most probably interacts with the ligand as well, although it may be shorter than the loops found in the V domains of the α, β, and δ chains.TRG V domain CDR3 loops in human and mouse are 1–12 and 4–11 amino acid long, respectively. This feature seems to be associated with a low expression of terminal deoxynucleotidyl transferase (TdT) during the TRG V-J rearrangements, and with the presence of short homology regions at the extremities of V and J genes that mediate direct recombination [34]–[36]. The TRG diversity may be further limited if the locus is organized in cassettes, as in mouse. In those cases TRG rearrangements occur within clusters of TRGV, TRGJ and TRGC genes which represent recombination units, and create mechanistic constraints on the TRGV-TRGJ rearrangements. This may also lead to rearrangements occurring at higher frequency between given TRGV and TRGJ and producing TRG chains with invariant junctions that are expressed even in the absence of the selection [37] Several of these specific features seems to be retained in fish [15], [17] as, for instance, the size of TRGV-TRGJ junction that appears to be shorter in the Japanese flounder, whereas the organization of the TRG locus with several V-J-C clusters is similar to the one observed in mouse [15]. In sea bass, the length of TRG CDR3-IMGT found in our sequenced clones varied between 9 and 15 amino acids (see Figure S1) and did not obviously follow the same trend as in mammals. In fact, the junction length spectratyping (Figure 3) showed that sea bass TRG CDR3 lengths are 8–18 AA (mostly 11–15), and TRG CDR3 length distribution is narrow, as already reported in the Japanese flounder and mammals. The conservation of this peculiarity, which has been associated to the direct antigen binding by γδ T cells (while αβ T cells are MH restricted [38]), may suggest that fish γδ T cells recognize their targets as their mammalian counterparts. Additionally, a significant number of CDR3 sequences (9 among 35) were found several times in the Japanese flounder, suggesting that the repertoire of these receptors may be strongly constrained [15].

Our results show that TRG CDR3 length distributions are generally not gaussian-like as usually seen for other Ag-specific receptors in naive individuals. This is reminiscent of what was reported for TRG rearrangements implicating TRVG 3, 5, 8 and 9 genes from human spleen [39, Figure 4C]. Such irregular profiles were even found in the thymus, while a large unbiased diversity is observed in this tissues for example for TRB [40], [54]. To our knowledge, TRG CDR3 length distributions have not been characterized from the thymus of other species. Hence, further studies will be necessary to determine if the thymic restriction of TRG repertoire is due to the particular rearrangement contraints of this isotype and represents a feature conserved through vertebrates. In any case, sea bass TRG CDR3 length spectratypes contains several peaks and show that the rearrangements expressed in thymus and gut are not restricted to one or two canonical sequences.

Furthermore, the spectratypes appeared to be different between thymus and gut of the same animal for a given V/C combination, which suggests that different repertoires are expressed in these tissues. To date, there is no evidence in fish for thymus-independent γδ-T cell differentiation in the gut. At this stage, it is therefore not possible to decide if these odd distributions are due to selection pressures exerted on the γδT cells in both thymus and gut, or if TRG rearrangements are strongly constrained at the recombination level, thus leading to skewed distribution in the thymus and, possibly, to tissue specific invariant TRG chain as observed in mouse skin and vaginal epithelium [37], [41].

Regarding cathe interaction with TRD chain, the molecular modeling of sea bass TRG chain and its comparison with human TRG chain suggest whose regions in the V-DOMAIN and in the C-DOMAIN would be involved (1hxm, Contact analysis in IMGT/3Dstructure-DB, http://www.imgt.org) [42]–[43]. Among these regions, the segments located in the V-domain are identical or with conservative peptide changes in the sea bass sequences Diclab1 and Diclab2, whereas regions in the C-Domain of Diclab1 and Diclab 2 show clear differences in the content of charged side chains (see fig. 2 and fig. 4b). In the C domain a high number of basic and acidic amino acids that may interact with other chains in the TR/CD3 complex are present. Overall, these data suggest that the structural features of TRG domains involved in TR γδ inter-chain interactions are conserved between fish and mammals.

The characterization of sea bass TRG expression in different tissues extends and confirms previous observations in carp and sea bass, [17], [29], [44]. The receptor appears to be predominantly expressed in thymus, where T cell lymphopoiesis occurs [45], and in mucosa. Investigating possible activities associated with TRG-bearing cells, our results show that in sea bass TRG expression in leukocytes can be modulated by poly I:C and, in turn, induction by poly I:C might indicate that IFN is involved. Indeed, human γδ T cells express more TLR3 mRNA than αβ T cells [46], and the TLR3 ligand enhance their *in vitro* activation via the stimulation of type I IFN production [47].

Interestingly, sea bass infection *in vivo* by a natural viral pathogen, the betanodavirus [30], induced significant modifications of the TRG expression in intestine and head kidney. These results are similar to observations reported in macaques after an *in vivo* infection with the simian immunodeficiency virus (SIV). In this model, the SIV infection resulted in a fast decrease of γδ T cell mRNA levels at mucosal sites, and in an increase at lymphoid sites, suggesting a rapid redistribution of γδ T cells after the infection [48]. In the same line, our results in sea bass may be interpreted as either a true down regulation and/or a migration of γδ T cells out of the tissue. Additionally, the TRG up regulation in the head kidney may be partly due to a higher transcription rate of the gene in activated cells. In helping to have new knowledge on this, future experiments the anti- sea bass T cell mAb DLT15 [44] will be employed in for the *in situ* labelling of immunoreactive cells during *in vivo* and *in vivo* stimulations.

In conclusion, the identification of TRG gives the possibility to investigate on the presence and functions of new T cell subsets in sea bass. In particular, our results indicate that sea bass γδ T cells are affected by the interferon response, and are probably involved in virus-induced immunity. Future studies will address the importance of these cells for the immunity to infections in fish.

## Methods

### Sea Bass TRG Chain Cloning and Sequence Analysis

Two primers (TRGF1∶5′- GAGGAACTGACCAGTGTAG -3′ and TRGR1∶5′- AGCAAGAGAGTCCACAGCA -3′) corresponding to highly conserved regions of known TRG genes were used in RT-PCR on total RNA extracted with Trisure (Bio-line) solution from the thymus of a juvenile sea bass (150 g of weight). The leukocytes from thymus were obtained following procedures already described [49]. RT-PCR was performed using Ready-To-Go RT-PCR Beads (GE Healthcare). For cDNA synthesis, 1 µg of total RNA and 0.5 µg of random primers [pd(N)_6_] were used in each reverse transcription reaction in a total volume of 50 µl. Reactions were conducted using the Mastercycler personal (Eppendorf). The cycling protocol was one cycle of 94°C for 5 min, 35 cycles of 94°C for 45 s, 54°C for 45 s, 72°C for 45 s, followed by one cycle of 72°C for 10 min. PCR products (15 µl) were visualised on 1% (w/v) agarose gels containing Gel Red using hyperladder IV (Bioline) as size marker. Controls for the presence of DNA contamination were performed using the RNA samples as template in the PCR cycle. DNA amplified by PCR was purified using the QIAquick Gel Extraction Kit (QIAgen), inserted into the pGEM-T Easy vector (Promega) and transfected into competent JM109 *Escherichia coli* cells. Plasmid DNA from at least ten independent clones was purified using the Wizard Plus SV Minipreps DNA Purification System (Promega) and sequenced using MWG DNA Sequencing Services. Sequences generated were analysed for similarity with other known sequences using the BLAST program [50].

A RACE-PCR was performed to obtain the complete TRG chain sequence. cDNA was synthesised from total thymus RNA with the First-strand cDNA Synthesis kit (GE Healthcare) following the manufacturer’s instructions. For 3′ RACE-PCR, cDNA was transcribed using an oligo-dT adaptor primer (5′-CTCGAGATCGATGCGGCCGCT_15_-3′). PCR was performed with the TRGF1 primer and the oligo-dT adaptor primer. For 5′ RACE-PCR, cDNA was transcribed from total RNA using the oligo-dT primer, treated with *E. coli* RNase H (Promega), purified using a PCR Purification Kit (QIAgen), and tailed with poly(C) at the 5′ end with terminal deoxynucleotidyl transferase (TdT, Promega). PCR was performed with TRGR1 primer and an Oligo-dG primer (5′-GGGGGGIGGGIIGGGIIG-3′). Sequencing and similarity searches were performed as described above. The expression of these sequences was confirmed by PCR using primers that amplify the complete coding sequence (data not shown).

The obtained TRG cDNA sequences were analysed for the presence of a signal peptide, using SignalP software [51], and of N- (with the NetNGlyc 1.0 Server) and O-linked glycosylation sites [52]. The TRG nucleotide and amino acid sequence were compared with counterparts in other vertebrate species with the EMBOSS Pairwise Alignment tool. Alignment of the sea bass TRG amino acid sequences to other known molecules from other species was carried out using MEGA 4.1 Software [53].

### CDR3 Length Spectratyping Analysis

The spectratyping of sea bass TRG CDR3 length was previously adapted for rainbow trout TRB [54]. A first amplification using a forward primer specific for a TRVγ sequence in combination with a reverse primer specific for a TRCγ sequence (see Table I for the primer combinations), was performed as follows: 1 µl cDNA was used as template for PCR1 using 0.4 mM of each dNTP, 0.4 µM of each primer (forward: TRG V, reverse: TRγC), and 0.025 u µl^−1^ of GoTaq DNA polymerase (Promega) in 1x reaction buffer with 2 mM of MgCl_2_ (95°C for 5 min; 40 cycles of 95°C 45 s, 60°C 45 s, 70°C 45 s; 70°C 10 min) (see Table I for primer sequences). This first PCR amplifies sequences for a given TRGV- TRGC combination but with different TRGJ content and diverse CDR3 lengths. In a second step, these PCR products are subjected to run-off reactions using 5′ 6-FAM- fluorescent internal, isotype TRGC-specific, reverse primers. Two µl of product of the first PCR were used as template using 0.4 mM of each dNTP, 10 pmoles of the fluorescent reverse primer, and 0.025 µg/ml of GoTaq DNA polymerase (Promega) in 1x reaction buffer with 2 mM of MgCl_2_ (95°C for 5 min; 5 cycles of 95°C 1 min, 60°C 1 min, 70°C 2 min; 70°C 10 min). Two µl of run-off product were mixed with 8 µl deionized formamide (Applied Biosystems) and 0.5 µl of the internal standard (GeneScanTM 500XL ROX, size standard, Applied Biosystems). Mix was denatured at 95°C for 5 min and placed on ice before analysis in an ABI 3730HT sequencer (Applied BioSystems) at GeT-PlaGe core facility, Toulouse, France. CDR3 length distributions were analyzed using GeneMapper (Applied BioSystems) and ISEApeaks software [55] extract and analyze spectratype data for each VH-C combinations. Each spectratype or profile is composed of several peaks (typically 4 to 10 for VH-Cμ and VH-Cδ, and 5 to 13 for VH-Cτ) separated according to their corresponding length of run-off products, spaced by 3 nucleotides as expected for in-frame transcripts.

### Three-dimensional Modeling of Sea Bass TRG Chain

Molecular modeling of sea bass TRG chain was performed in agreement with well established procedures, previously described in our works [56]–[58]. The experimental model from PDB data bank related to the Human Vγ9Vδ2 T cell receptor (code 1HMX, chain B), was chosen as the template for the comparative modeling strategy. The percentage of sequence identity (27%) required special care for the alignment of the sequences, in order to verify its quality before the successive modeling procedures. The alignments obtained by different free available software (BLAST, EMBOSS Align, ClustalW) were compared and, finally, the alignment with the right position of the cysteine residues involved in the disulphide bridges was chosen. Modeller9v5 (http://salilab.org/modeller/) was used to create models of both the identified sea bass TRG chain sequences, after the removal of the 1–19 segment (signal peptide, not present in the template structure). The best model was selected by verifying the quality of the models in terms of stereochemistry, by using PROCHECK software [59] and in terms of energy, by using PROSA-web server [60]. Further analysis of the model was performed with tools for the visualization and evaluation of structural parameters.

### *In vitro* and *in vivo* TRG Expression Analysis

The *in vitro* TRG expression was studied on head kidney (HK) leukocytes obtained from five sea bass juveniles as already described [49]. Fish were purchased from a local fish farm (Civita Ittica Srl, Civitavecchia, Italy), lethally anaesthetised with phenoxyethanol (100 ppm), and organs removed and placed in cold HBSS. Head kidney and gut leukocytes were adjusted to 1×10^5^ cells/ml and incubated at 18°C for 6 h and 24 h with 20 µg/ml of poly I:C (Sigma). Leukocytes from kidney and the intestinal tract (whole, excluding stomach) were obtained as previously described [30]. The control was stimulated with PBS only at the same time points. Total RNA was isolated with Trisure (Bio-line) after stimulation periods, resuspended in DEPC-treated water and used for real-time quantitative PCR without pooling the samples coming from the different flasks. Controls for the presence of DNA contamination were performed with RT-PCR using β-actin primers that span an intron (ACTFOR: 5′ –ATGTACGTTGCCATCC- 3′; ACTRV: 5′-GAGATGCCACGCTCTC- 3′).

The *in vivo* TRG expression was studied on head kidney (HK) and gut leukocytes obtained from five sea bass juveniles experimentally infected with betanodavirus (nervous necrosis virus, NNV) [30]. Sea bass samples for TRG expression were from *in vivo* experiments previously performed and already described in details [30]. From each individual fish the head kidney and gut were removed after infection with NNV at hours: 0, 6, 24, 72. The tissue was immersed in 1 ml of Trizol, labelled, immediately frozen in liquid nitrogen and stored at −80°C until further use. Total RNA was extracted as described before.

For reverse transcription, the BioScript RNase H minus (Bioline) enzyme was used with the with the protocol described in [56]. The expression level of the target genes was determined with a Mx3000P^TM^ real time PCR system (Stratagene) equipped with version 4.0 software and using the Brilliant SYBR Green Q-PCR Master Mix (Stratagene) following the manufacturer’s instructions, with ROX as internal passive reference dye. Specific PCR primers were designed for the amplification of about 200 bp products from the constant region of sea bass TRG (TRGF2∶5′ -CTGCTGTGTGTGGCCTCAGAC- 3′ and TRGR2∶5′ –GTGCTGGACGGAGCAGTGGTA- 3′) and sea bass ribosomal RNA 18S (RIBFR: 5′ – CCAACGAGCTGCTGACC- 3′ and RIBRV: 5′ –CCGTTACCCGTGGTCC- 3′), used as a house-keeping gene. Ten ng of cDNA template were used in each PCR reaction. The PCR conditions were 95°C for 10 min, followed by 35 cycles of 95°C for 45 s, 52°C for 45 s and 72°C for 45 s. Triplicate reactions were performed for each template cDNA and the template was replaced with water in all blank control reactions. The analysis was carried out using the endpoints method option of the Mx3000P^TM^ software that causes the collection of the fluorescence data at the end of each extension stage of amplification. A relative quantitation has been performed, comparing the levels of the target transcript to a reference transcript (calibrator: the time 0 control for the stimulations and the not treated specimens for the virus infection). A normalizer target (the ribosomal RNA transcript) is included to correct for differences in total cDNA input between samples. The results are expressed as the mean ± SD and the differences from the controls have been considered significant if p<0.05 using a statistical analyses performed by the one-way ANOVA followed by the Bonferroni test. The real-time PCR products from the different experiments were examined by agarose gel electrophoresis to investigate their specificity and size.

### Ethic Statements

Fish for *in vitro* experiments were purchased from a local fish farm (Civita Ittica Srl, Civitavecchia, Italy), lethally anaesthetised with phenoxyethanol (100 ppm), and organs removed and placed in cold HBSS.

The samples of the *in vivo* work came from experiments described in Scapigliati et al., 2010. Experiments were performed at the research facilities of CIFPA El Toruño, Cadiz, Spain, during the EU funded project “IMAQUANIM”, and were approved by the IMAQUANIM consortium.”

## Supporting Information

Figure S1 **Multiple alignment of TRG sequences with different V and C sequences from RACE cloning or genomic contigs (noted .Gen).**
(TIF)
