# Supplementary figures and images for: Diversity, Molecular Characterization and Expression of T Cell Receptor γ in a Teleost Fish, the Sea Bass (Dicentrarchus labrax, L)
Source: PLoS One. 2012 Oct 25;7(10):e47957. doi: 10.1371/journal.pone.0047957 (PMC3485050; doi:10.1371/journal.pone.0047957)

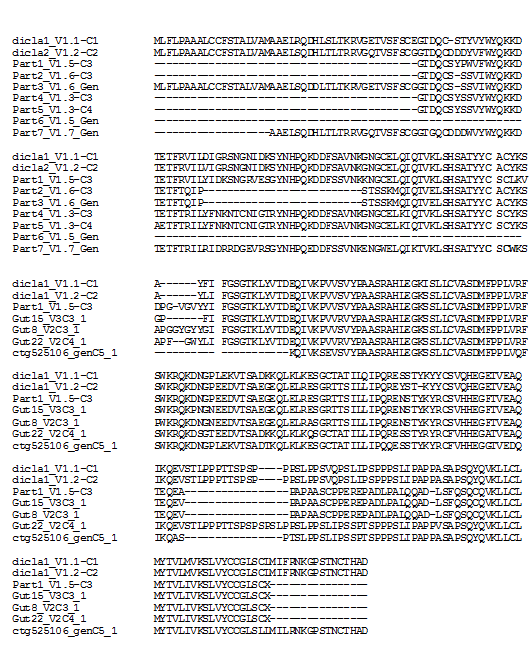

Supplement: Figure S1 — Multiple alignment of TRG sequences with different V and C sequences from RACE cloning or genomic contigs (noted .Gen). (TIF) [file pone.0047957.s001.tif]
